# Mental Health Literacy Among Future Health and Education Professionals: Validity Evidence of the Spanish Version of the Mental Health Literacy Questionnaire for Young Adults

**DOI:** 10.3390/ejihpe16100149

**Published:** 2026-09-25

**Authors:** Jairo Rodríguez-Medina, Carlota Alcover Van de Walle, Paula San Martín González, Clara González-Sanguino

**Affiliations:** 1 Department of Pedagogy, University of Valladolid, 47011 Valladolid, Spain; jairo.rodriguez.medina@uva.es; 2 Social Determinants and Health Education Research Group (SDHEd), Hospital del Mar Research Institute, 08003 Barcelona, Spain; carlota.alcover.vandewalle@hmar.cat; 3 Hospital del Mar Nursing School (ESIHMar), Universitat Pompeu Fabra-Affiliated, 08003 Barcelona, Spain; 4 Department of Nursing, Pontifical University of Salamanca, 37002 Salamanca, Spain; psanmartingo@upsa.es; 5 Department of Psychology, University of Valladolid, 47011 Valladolid, Spain

**Keywords:** mental health, health literacy, psychometrics, education students, nursing students

## Abstract

Mental health literacy (MHL) plays a crucial role in the early identification, management, and prevention of mental health problems, especially among young adults. For future health and education professionals, robust MHL is a foundational component of clinical readiness, ensuring they are adequately prepared to recognize early signs of psychological distress, mitigate stigma, and facilitate timely referrals in healthcare and community settings. This study aimed to examine the internal structure and psychometric properties of the Mental Health Literacy Questionnaire for Young Adults (MHLq-YA) within a Spanish university population. An initial sample of 425 university students enrolled in Nursing and Education programs was recruited. Following data screening for invariant and aberrant responding, the final analytical sample comprised 416 participants. Confirmatory Factor Analysis (CFA) supported a four-factor structure (Knowledge, Stereotypes, Help-Seeking, and Self-Help) with an adequate and structural fit to the data, supporting its multidimensionality with an adequate model fit (CFI = 0.949, TLI = 0.944, RMSEA = 0.068). Evidence of convergent and discriminant validity was observed in three of the four dimensions, while the relationship between Knowledge and Self-Help warranted theoretical interpretation. Convergent validity was supported for three dimensions (Stereotypes, Help-Seeking, and Self-Help), whereas the Knowledge dimension showed comparatively weaker convergent validity (AVE = 0.436). Discriminant validity was supported for all factor pairs under the HTMT criterion, although the stricter Fornell–Larcker criterion was not met for the Knowledge–Self-Help pair, reflecting a conceptual and empirical overlap between these two dimensions that is discussed as a structural limitation of the model. Item Response Theory (IRT) analyses revealed high measurement precision in the lower and moderate ranges of the latent traits, particularly for the Help-Seeking and Self-Help subscales. Ceiling effects and cross-loadings in specific items were resolved through a stepwise model re-specification. The final 26-item version demonstrated robust psychometric performance. These findings support the MHLq-YA as a valid and reliable tool for assessing mental health literacy in academic settings. Crucially, it allows educators to identify gaps in clinical preparedness before students interact with actual patients or vulnerable populations. Ultimately, these results inform targeted interventions to improve mental health awareness, help-seeking behavior, and the overall quality of future clinical care provided by university students.

## 1. Introduction

Mental health problems represent one of the leading causes of disability and functional impairment in young populations, affecting both personal well-being and academic and professional performance (WHO, 2023). The prevalence of disorders such as anxiety and depression among university students has significantly increased over the past decade, positioning mental health as a priority on both educational and healthcare agendas (Healthy Minds Study, 2025; Auerbach et al., 2018). This scenario underscores the need for preventive and intervention strategies aimed at reducing the impact of these issues during critical formative stages.

In this context, mental health literacy (MHL) is defined as the set of knowledge and skills that facilitate the identification, understanding, and management of psychological problems, as well as access to appropriate treatment resources (Jorm, 2020). MHL encompasses essential components such as knowledge of symptoms and causes, stigma reduction, willingness to seek help, and the adoption of self-care strategies (Kutcher et al., 2016). Adequate MHL not only promotes early detection and effective intervention but also contributes to the promotion of psychological well-being in educational and healthcare settings.

The relevance of this issue is amplified when considering the role of future professionals in health and education, who act as key agents in the prevention and management of mental health problems in both clinical and educational contexts. Low levels of MHL in these groups may limit the effectiveness of interventions, perpetuate stigmatizing attitudes, and hinder support capacity (McCormack et al., 2025; Tullius et al., 2025). In contrast, higher MHL levels are associated with greater willingness to seek help, more positive attitudes toward mental health, and better psychological well-being outcomes (Sergeyev et al., 2025; Gorczynski & Sims-Schouten, 2024). Therefore, the assessment and improvement of MHL in these groups constitute a priority for research and practice (Khaleel Younis, 2025).

Various tools have been developed to assess MHL in young adults. In Europe, in a context apparently similar to the Spanish one, the Mental Health Literacy Questionnaire for Young Adults (MHLq-YA) stands out. It was originally developed and validated in Portugal (Dias et al., 2018), and the original instrument comprises 29 items distributed across four dimensions: knowledge of mental health problems, erroneous beliefs/stereotypes, first-aid skills and help-seeking behavior, and self-help strategies. Since its creation, the MHLq-YA and its brief 16-item version (MHLq-SVa) have demonstrated robust psychometric properties across diverse cultural contexts. A validation study across six countries (China, India, Indonesia, Portugal, Thailand, and the United States) confirmed its four-dimensional construct validity and high internal consistency (Campos et al., 2022). More recently, these findings were replicated in a multicenter study spanning eleven additional countries (including Brazil, Germany, and Poland), further supporting its cross-cultural reliability, measurement invariance, and utility as an effective MHL assessment tool for young adults worldwide (Costa et al., 2025).

Similarly, in the Middle Eastern and Eastern European context, Esen (2025) carried out the Turkish adaptation of the MHLq-SVa. The psychometric analysis corroborated that the tool presents excellent indices of reliability and convergent validity in the young adult population of Turkey. Crucially, this study supported the validity of the questionnaire by retaining four distinct sub-dimensions: knowledge about mental health problems, stereotypes, help-seeking behavior, and self-help strategies. The authors concluded that these specific metric characteristics allow for the precise identification of deficiencies in symptom recognition and stigmatizing prejudices in this age group, thereby facilitating the targeted evaluation of educational programs in mental health.

In the European context, the validation of the instrument has continued to expand. In Italy, Pastore et al. (2025) analyzed the full version of the MHLq-YA in young adults, concluding that the scale possesses high internal consistency and structural validity. Specifically, their study confirmed the original four-factor structure, which encompasses: knowledge of mental health problems, erroneous beliefs and stereotypes, first-aid skills and help-seeking, and self-help strategies. Their results demonstrated that the questionnaire functions reliably in capturing these key dimensions of MHL, highlighting its utility as a diagnostic tool prior to designing preventive interventions in Italian university settings.

In Spain, recent advances in MHL measurement have focused predominantly on adolescent populations. For instance, instruments such as the EspaiJove.net Mental Health Literacy (EMHL) test (Casañas et al., 2020) and the Spanish version of the MHLq for adolescents (MHLq-E) have been recently validated to assess knowledge, stigma, and help-seeking specifically in secondary school students aged 12 to 16 (González-Sanguino et al., 2025). The psychometric evaluation demonstrated that the MHLq-E possesses satisfactory internal consistency and strong construct validity within this demographic. Factorial analyses confirmed a multidimensional structure that aligns with the core of 3 components, specifically capturing dimensions such as knowledge about mental disorders, stigmatizing attitudes and stereotypes, and help-seeking behavioral intentions. The authors concluded that the MHLq-E is a highly reliable and culturally appropriate tool for measuring mental health literacy in Spanish-speaking adolescents.

Focused on Spanish-speaking adults, other tools like the Universal Mental Health Literacy Scale (UMHL) have been adapted (Campo-Arias et al., 2025), but they have primarily been validated in Latin American contexts rather than in Spain, and lack specificity for the young adult stage. The UMHL had 17 items, and the structural analysis identified two main subscales: attitudes and knowledge. The attitudes subscale was further divided into two dimensions—help-seeking and stigma/discrimination—while the knowledge subscale comprised a single dimension. Although the overall dimensionality showed an acceptable fit, the study highlighted notable limitations regarding internal consistency. Specifically, while the help-seeking and knowledge domains were appropriate, the stigma/discrimination dimension exhibited low reliability (Cronbach’s alpha = 0.45), indicating a need for further refinement.

Consequently, there is currently a lack of self-report questionnaires rigorously validated in Spain that evaluate the multidimensional nature of MHL explicitly in young adult and university populations.

Consequently, a critical methodological gap remains within the Spanish academic and clinical context: the absence of a rigorously validated, multidimensional self-report measure specifically calibrated for the psychosocial and developmental realities of young adults. Addressing this deficiency is of paramount importance for university students training as future health and education professionals, given that their own MHL will directly influence their prospective clinical and pedagogical efficacy.

To bridge this gap in the literature, the present study aims to culturally adapt and psychometrically validate the Spanish version of the Mental Health Literacy Questionnaire for Young Adults (MHLq-YA). Specifically, this research evaluates the psychometric properties of the Spanish-translated instrument within a cohort of university students enrolled in education and nursing degree programs (N = 416). The primary objectives are: first, to examine the construct validity of the Spanish adaptation by corroborating its underlying dimensional structure through Confirmatory Factor Analysis (CFA); and second, to execute a robust, item-level evaluation utilizing Item Response Theory (IRT). Integrating IRT allows for a highly precise, granular understanding of individual item performance across the continuum of the latent mental health literacy trait. Ultimately, the validation of this instrument seeks to provide a reliable, culturally anchored metric to accurately assess mental health literacy in this critical demographic, thereby establishing an empirical foundation for the design and evaluation of targeted preventive interventions within Spanish higher education institutions.

## 2. Methods

### 2.1. Design

A descriptive cross-sectional study was conducted with university students from various Spanish faculties enrolled in undergraduate Nursing and Education programs (Early Childhood and Primary Education). Participants were recruited in their regular classrooms by their professors. After a brief explanation of the study, students who voluntarily wished to participate completed the assessment protocol online via computer, tablet, or smartphone. The protocol lasted approximately 15 min. Participation was anonymous, and informed consent was obtained in accordance with ethical guidelines. The inclusion criteria for the study were: (a) being actively enrolled in an undergraduate Nursing or Education program, and (b) being 18 years of age or older. Exclusion criteria included refusing to provide informed consent or failing to complete the required psychometric scales. To concretely enforce these criteria, the online survey platform was configured with mandatory screening questions. If a participant did not electronically check the informed consent agreement or indicated being under 18 years old, the survey automatically terminated without collecting further data. Additionally, during the data curation phase, responses with significant missing data were systematically excluded. This study is part of the “Stigma and Discrimination as Factors of Vulnerability in Childhood,” founded by la Caixa (FS23-IB), and the MENTALSED project, funded by MICIU/AEI/10.13039/501100011033 and by ERDF/EU and was approved by the Ethics Committee of the University of Valladolid prior to its implementation.

### 2.2. Participants

Participants were selected using convenience sampling during the 2024–2025 academic year. An initial sample of 425 university students enrolled in Nursing (n = 206, 48.47%) or Education programs, including Primary Education (n = 169, 39.76%) and Early Childhood Education (n = 50, 11.76%), was recruited. As detailed in the subsequent data screening section, following the removal of invariant and aberrant response patterns, the final analytical sample retained for psychometric testing comprised 416 valid cases. The mean age of the sample was 20.32 years, with a clear majority of women (76.5%). The sociodemographic characteristics can be seen in detail in Table 1.

Sociodemographic characteristics were assessed using ad hoc questions on gender (male, female), age, field of study (primary education, early childhood education, nursing), year of study (1–4), province of residence, size of place of residence (city with more than 500,000 inhabitants, between 100,000 and 500,000 inhabitants, between 20,000 and 100,000 inhabitants, less than 20,000 inhabitants), previous contact with people with mental health problems (yes, no), religion (Catholic, other, non-believer, unspecified), and political ideology (centrist, conservative, progressive, unspecified).

### 2.3. Variables and Instruments

In addition to the main psychometric instruments, basic sociodemographic data (e.g., age, gender, and degree program) were collected. The inclusion of these variables served a dual methodological purpose. First, it allowed for the accurate characterization of the sample and ensured that the nursing and education student cohorts were demographically comparable, thereby justifying their joint inclusion in the validation analyses. Second, gathering this information establishes a necessary foundation for future research, enabling subsequent comparative analyses of mental health literacy levels across specific sociodemographic subgroups.

Mental health literacy was assessed using the Spanish version of the Mental Health Literacy Questionnaire for Young Adults (MHLq-YA; Dias et al., 2018), an instrument originally developed and validated for the university-aged population in Portugal. Since its creation, the MHLq-YA has been culturally and linguistically adapted for other age groups and contexts, including an adolescent version validated in Spanish (MHLq-E; González-Sanguino et al., 2025) and short forms validated across several countries (Campos et al., 2022).

The original MHLq-YA comprises 29 items rated on a 5-point Likert-type scale (1 = strongly disagree to 5 = strongly agree), organized into four theoretical dimensions: (1) Knowledge of mental health problems, assessing knowledge about the symptoms and causes of mental health problems; (2) Stereotypes, assessing misconceptions and stigmatizing attitudes toward mental health; (3) Help-Seeking, assessing first-aid skills and the ability to seek help for oneself or others; (4) Self-Help, assessing strategies for managing one’s own mental health problems.

Six items are reverse-scored prior to computing dimension scores (items 6, 10, 13, 15, 23, and 27); in all cases, higher scores denote greater mental health literacy.

In its original Portuguese validation, the MHLq-YA showed good internal consistency (total scale α = 0.84; subscale α = 0.74, 0.72, 0.71, and 0.60 for Knowledge, Stereotypes, Help-Seeking, and Self-Help, respectively; Dias et al., 2018). The instrument has since been adapted into several languages, including Turkish, Chichewa, and Chinese (Günaydin et al., 2023; Jumbe et al., 2023; Pribadi et al., 2023; Wu et al., 2023), consistently replicating its four-dimensional structure.

### 2.4. Data Analysis

The data processing was conducted in several phases. First, a data cleaning process was carried out, and subsequently, evidence of validity and reliability was obtained through Confirmatory Factor Analysis (CFA) and Item Response Theory (IRT) models.

#### 2.4.1. Preliminary Data Analysis and Sample Screening

Prior to the main analyses, a systematic data screening and cleaning protocol was implemented. The initial sample, after filtering to include only undergraduate students in the target degree programs (Nursing, Primary Education, and Early Childhood Education), consisted of 425 participants.

The screening process proceeded in several steps to ensure data validity. First, to detect invalid response patterns, an initial check for invariant responding was conducted. Using the intra-subject standard deviation across the 29 MHLq-YA items, three participants who provided the same response to all items were identified. As this pattern indicates non-engagement with the questionnaire content, these cases were excluded, reducing the sample to N = 422.

Next, the sample was assessed for the presence of influential cases by identifying multivariate outliers using Mahalanobis distance (D^2^). This analysis, conducted on the 422 participants, identified 32 participants (7.58%) as potential multivariate outliers (*p* < 0.001), with a maximum D^2^ value of 154.02. In accordance with methodological recommendations for caution against the arbitrary removal of outliers (Kline, 2023), it was decided to retain these participants. Their potential influence on the results will be appropriately managed through the use of robust estimation methods (e.g., WLSMV) in subsequent structural equation modeling analyses.

Finally, to detect inconsistent or aberrant response patterns that might not be captured by Mahalanobis distance, a Person-Fit Analysis was performed. The standardized log-likelihood statistic (*l*z) was computed for each of the 422 participants, a method designed to assess the appropriateness of response vectors within polychotomous item response models (Drasgow et al., 1985). This index is effective at detecting unexpected response patterns often associated with careless or random responding (Niessen et al., 2016). Figure 1 displays the density histogram for the resulting *l*z scores. The distribution approximates normality, although with a slight negative skew, indicating that most participants responded in a manner consistent with the measurement model. Following a strict exclusion criterion (*l*z ≤ −3), six participants (1.42%) exhibiting highly improbable response patterns were identified and removed. For informational purposes, the figure also shows a more lenient cutoff (*l*z ≤ −1.65), which would have resulted in the exclusion of 26 participants (6.16%).

Following this comprehensive screening process, the final sample utilized for the main analyses was established at N = 416. This sample size is considered robust and adequate for conducting the planned statistical analyses, including Confirmatory Factor Analysis (CFA), measurement invariance testing, and latent mean comparisons.

#### 2.4.2. Validity Evidence Based on Internal Structure

To evaluate the factorial structure of the MHLq-YA, two theoretical models were specified and compared through CFA on the final sample (N = 416): a unidimensional model and the theoretical model of four correlated factors (Knowledge, Stereotypes, Help-Seeking, and Self-Help).

To obtain evidence of convergent validity, the following criteria were assessed: (a) whether the factor loadings (λ) of all indicators were statistically significant and substantial (greater than 0.50); and (b) whether the Average Variance Extracted (AVE) for each factor exceeded 0.50.

To assess discriminant validity, two main approaches were employed (Hair et al., 2019). First, the Fornell–Larcker criterion was applied, checking whether the square root of the AVE for each factor was greater than the correlation of that factor with the others. Second, the Heterotrait–Monotrait ratio (HTMT) of correlations was calculated, using a strict threshold of <0.85 to confirm discrimination between factors (Henseler et al., 2015).

All models were estimated using the Diagonally Weighted Least Squares (DWLS) estimator, robust for ordinal data, on the polychoric correlation matrix. Model fit was assessed using the following indices and cutoff criteria: CFI and TLI (≥0.95), RMSEA (≤0.06) with its 90% CI, and SRMR (≤0.08) (Hu & Bentler, 1999). The comparison between nested models was based on the chi-square difference test.

#### 2.4.3. Psychometric Item Analysis: Item Response Theory (IRT)

To deeply analyze item performance, a Multidimensional Graded Response Model (GRM) was fit to the data from the final sample. This model was chosen as it is suitable for ordered polytomous items (Likert scales) and because of its ability to model the structure of four correlated factors in a manner coherent with CFA. Based on this model, the following aspects were analyzed: (a) Item parameters: The discrimination parameter of each item (its ability to differentiate between participants with different levels of the latent trait), and the difficulty or threshold parameters, which indicate the point on the trait continuum where it is most likely for a respondent to transition from one response category to the next. (b) Scale information: Test Information Functions (TIFs) were generated and analyzed for each of the four subscales, in order to determine the range of the construct (low, medium, or high levels of literacy) where the questionnaire measures with greater or lesser precision.

Given the ordinal 5-point Likert scale, the GRM typically estimates four thresholds ($\beta_{1}$ to $\beta_{4}$). However, preliminary descriptive analyses revealed zero frequencies in the lowest response categories for several items due to severe ceiling effects. These unobserved categories were not artificially collapsed; rather, the GRM naturally omitted their transitions. The resulting threshold parameters were manually aligned in the results tables to accurately reflect their true empirical category transitions (e.g., $\beta_{2}$ representing the transition from category 2 to 3).

### 2.5. Reliability Analysis

Given the ordinal nature of the Likert-type items and the use of the DWLS estimator, internal consistency was calculated using the Categorical Omega ($\omega_{cat}$) proposed by Green and Yang (2009). Unlike simple ordinal omega, Categorical Omega incorporates both the polychoric factor loadings and the categorical thresholds to estimate the reliability of the observed discrete sum scores, providing a more realistic, albeit conservative, measure of internal consistency.

All analyses were carried out using R software, version 4.3.1 (R Core Team, 2023), primarily employing the lavaan package (Rosseel, 2012) for CFA and mirt for the IRT models.

## 3. Results

To evaluate the internal structure of the MHLq-YA in the study sample (N = 416), three theoretical models were tested and compared through Confirmatory Factor Analysis (CFA). Table 2 presents the fit indices for each model and the differences between them.

Model 1 (Unidimensional), which assumes that all items load onto a single general factor, showed poor fit to the data, with values on the indices failing to meet commonly accepted thresholds (CFI = 0.834, TLI = 0.821, RMSEA = 0.167, SRMR = 0.242). This result allows for rejection of the unidimensionality hypothesis and confirms the multidimensional nature of the construct.

Both Model 2 (Four Correlated Factors) and Model 3 (Hierarchical) represented a substantial improvement in fit compared to the unidimensional model. Model 2 yielded the following indices: χ^2^(371) = 3402.627, CFI = 0.884, TLI = 0.873, RMSEA = 0.140, and SRMR = 0.192. When comparing Model 3 (Hierarchical) to Model 2 (used as the reference), a marginal improvement in fit was observed. The indices for the hierarchical model were slightly higher (CFI = 0.885, TLI = 0.875) or lower (RMSEA = 0.139), but the differences were minimal (CFI = 0.001, TLI = 0.002, RMSEA = −0.001). These changes do not indicate a meaningful improvement in the fit of the hierarchical model over the four correlated factors model.

In conclusion, although none of the proposed models achieves excellent fit according to conventional cutoff criteria in this sample, the data clearly support a multidimensional structure over a unidimensional one. Both the four-factor correlated model and the hierarchical model offer very similar fit and are substantially superior to the unidimensional model.

Initial evaluation of the 29-item measurement model revealed specific sources of misspecification. First, Item 21, theoretically assigned to the Stereotypes factor, exhibited an inadequate standardized factor loading (β = 0.252), failing to meet the minimum threshold for indicator reliability, and was thus removed. Subsequent analysis of the Modification Indices (MIs) highlighted several areas of significant tension. Item 27r (“A person with schizophrenia may see and hear things that no one else sees or hears”), theoretically assigned to the Knowledge factor, presented extremely high MIs suggesting strong cross-loadings on the Stereotypes (MI = 1977.307) and Self-Help (MI = 1824.033) factors. This indicates that the item does not measure a unique construct and introduces a substantial amount of noise and misfit into the model.

Additionally, Items 11 (“If someone close to me developed a mental disorder, I would listen to them without judging or criticizing”) and 14 (“The earlier mental disorders are identified and treated, the better”), both assigned to the Stereotypes factor, also exhibited high MIs, suggesting significant cross-loadings onto other factors (e.g., i11 on Knowledge and Self-Help; i14 on Knowledge and Help-Seeking).

Based on this diagnostic, the model was re-specified in two steps. First, the two items (i15r, i21 and i27r) generating the most severe anomalies (massive cross-loadings) were removed because their empirical misfit reflected genuine conceptual misalignments. Specifically, Item 27r describes a valid clinical diagnostic criterion, making its reversed scoring theoretically contradictory within the Knowledge factor; similarly, Items 15r (an epidemiological misconception) and 21 (low self-efficacy) conceptually diverged from the stigmatizing attitudes meant to be captured by the Stereotypes dimension. This refined model (Model 4) resulted in a substantial improvement in fit (CFI = 0.925, TLI = 0.918, RMSEA = 0.110), confirming the problematic nature of those items.

Finally, a theoretical re-specification was performed based on the content of the two items that continued to show misfit. Item 14 (“The earlier mental disorders are identified and treated, the better”) was reassigned from Stereotypes to Knowledge, as its content evaluates knowledge about best practices in mental health. Similarly, Item 11 (“If someone close to me developed a mental disorder, I would listen to them without judging or criticizing”) was reassigned from Stereotypes to Help-Seeking, as it describes a psychological first aid skill.

This final re-specified model (Model 5), composed of 26 items, showed a reasonably good fit to the data: χ^2^(293) = 917.72, CFI = 0.948, TLI = 0.943, RMSEA = 0.072, SRMR = 0.077. Although CFI and TLI values remain marginally below the strict 0.95 threshold, the model is theoretically sound, parsimonious, and presents an adequate empirical fit for a complex multidimensional structure. Therefore, it was selected as the final factorial structure.

Additionally, a sensitivity analysis was conducted to evaluate the structural impact of the multivariate outliers previously identified via Mahalanobis distance. When the 26-item CFA was re-estimated excluding these cases (yielding n = 387), the model demonstrated a substantial improvement in fit ($\chi^{2}$(293) = 806.61, CFI = 0.984, TLI = 0.982, RMSEA = 0.067, SRMR = 0.076). However, because the primary analytical sample (N = 416) already achieved adequate fit, and these atypical profiles likely reflect genuine ecological variations and valid extremes in MHL within the student population, rather than measurement error or careless responding. Retaining them preserves the natural variance of the sample; conversely, removing them would artificially restrict this variance and inflate the scale’s psychometric performance, thereby compromising the ecological validity of the validation process.

Table 3 presents the unstandardized factor loadings (B), standard errors (SE), z-statistics, standardized factor loadings (β), and the explained variance for each item (R^2^).

All items presented standardized factor loadings (β) that were statistically significant (*p* < 0.001) and substantial on their theoretical factors. Most loadings exceeded the value of 0.60, and none were below 0.33, which provides strong evidence of convergent validity: the items are good indicators of the latent constructs they are intended to measure. The Stereotypes factor showed the most consistently high loadings (β > 0.90), indicating strong cohesion among its items.

The reliability of each individual item can be assessed through its coefficient of determination or explained variance (R^2^). This value indicates the proportion of the item’s variance explained by its latent factor, with the remaining portion attributable to measurement error. Higher R^2^ values indicate greater indicator reliability.

In general, the items in the final model demonstrated adequate reliability. Notably, the items from the Stereotypes factor (i06r, i10r, i13r, i23r) had R^2^ values consistently above 0.82, indicating that more than 82% of their variance is explained by the factor, reflecting excellent reliability. Items such as i02 (Knowledge) and i08 (Help-Seeking) also showed high reliability (R^2^ > 0.70).

On the other hand, some items exhibited more modest reliability. Specifically, items i22 (R^2^ = 0.115) and i03 (R^2^ = 0.189) from the Knowledge factor, and item i04 (R^2^ = 0.356) from Help-Seeking showed the lowest explained variance values. Although their factor loadings are statistically significant and theoretically coherent, these results suggest they are the least precise indicators of their respective factors within the model. Nevertheless, the overall reliability of most items is moderate to high, supporting the overall quality of the measure.

Figure 2 presents the path diagram of the final four-factor correlated model for the Mental Health Literacy Questionnaire for Young Adults (MHLq-YA), based on the final sample (N = 416). In the diagram, the four latent constructs (circles/ovals)—Knowledge (Cnc), Stereotypes (Est), Help-Seeking (BsA), and Self-Help (Aty)—are shown, as well as the 26 observed variables (rectangles) that serve as their indicators.

The unidirectional arrows from latent factors to items represent the standardized factor loadings (β). As shown, all loadings are positive, substantial, and statistically significant, indicating that the items are good indicators of their respective factors. For example, items such as i23 (β = 0.94) and i10 (β = 0.93) are very strong indicators of the Stereotypes factor, while items such as i22 (β = 0.34) contribute more modestly to the Knowledge factor.

The curved bidirectional arrows between latent factors represent their correlations. Positive and moderate correlations were observed between Self-Help and Knowledge (0.82), and between Self-Help and Help-Seeking (0.60), suggesting that these constructs are related but distinct. Negative correlations, such as those between Stereotypes and the other three factors (−0.16, −0.18, −0.15), are theoretically consistent, indicating that higher levels of stereotypes are associated with lower scores in knowledge, help-seeking, and self-help. The moderate magnitude of these correlations provides evidence of discriminant validity among the four factors.

Finally, the small curved arrows pointing to each item (observed variables) represent the residual variance or measurement error of each indicator; that is, the portion of the item’s variance not explained by the latent factor. Lower values, such as 0.12 for item i23, indicate that the factor explains a large portion of the item’s variance (approximately 88%), reflecting high indicator reliability.

To complement this analysis, the reliability of each dimension was evaluated using McDonald’s Omega coefficient, which is a robust estimator of internal consistency. Based on the final 26-item model parameters, the Categorical Omega ($\omega_{cat}$) coefficients for the observed scores showed good to excellent internal consistency: Knowledge = 0.839; Stereotypes = 0.934; Help-Seeking = 0.849; and Self-Help = 0.750. These values confirm that the scale produces highly reliable observed scores.

To provide a descriptive summary of the overall instrument, global reliability metrics were calculated for the full 26-item scale. To prevent the artificial deflation of structural reliability, the four reverse-valenced Stereotypes items were mathematically inverted to align all indicators toward the positive pole of mental health literacy. Based on the polychoric correlation matrix, the overall instrument demonstrated excellent internal consistency, yielding a global ordinal Alpha of 0.920 and a global categorical Omega of 0.929. Nevertheless, given the empirically confirmed multidimensional structure of the MHLq, researchers are encouraged to prioritize the specific subscale scores to capture the distinct conceptual nuances of mental health literacy.

Finally, the analysis of the Average Variance Extracted (AVE) yielded more nuanced results. The factors Stereotypes (AVE = 0.855), Self-Help (AVE = 0.631), and Help-Seeking (AVE = 0.563) clearly exceeded the recommended threshold of 0.50, indicating that more than 50% of the variance of their indicators is captured by the latent construct. This provides strong evidence of convergent validity for these three dimensions.

However, the Knowledge factor (AVE = 0.436) did not reach the criterion, suggesting that although it is a reliable dimension, its indicators share a considerable amount of measurement error variance. Overall, the results confirm the reliability of all four dimensions. Strong evidence of convergent validity was obtained for the Stereotypes, Self-Help, and Help-Seeking factors, while the evidence for the Knowledge factor is more moderate.

To assess discriminant validity among the four factors of the final model, three increasingly demanding approaches were applied.

First, 95% confidence intervals for the standardized correlations between the latent factors were examined (Anderson & Gerbing, 1988). As shown in Table 4, the upper limit of all confidence intervals was clearly below 1.0, providing initial evidence that the four factors, although related, are empirically distinct.

The analysis of the magnitude of the correlations (r) revealed a very high association between the factors Knowledge and Self-Help (r = 0.822), suggesting considerable conceptual overlap between the two constructs. The correlations among the other positive dimensions of mental health literacy were moderate to high (Knowledge and Help-Seeking, r = 0.655; Help-Seeking and Self-Help, r = 0.600).

As expected from a theoretical standpoint, the Stereotypes factor showed negative and low-magnitude correlations with the other three factors (ranging from –0.151 to –0.183), indicating that greater stereotypical beliefs are associated with lower scores on the adaptive dimensions of mental health literacy.

As a second, more demanding approach, the Fornell and Larcker (1981) criterion was applied. This method establishes that, in order for discriminant validity to be confirmed, the Average Variance Extracted (AVE) of each construct (values on the diagonal of Table 5) must be greater than the shared variance (the squared correlation) with any other construct in the model.

The results presented in Table 5 show that this criterion was met for most relationships. Specifically, the factors Stereotypes (AVE = 0.855) and Help-Seeking (AVE = 0.563) demonstrate clear discriminant validity, as their AVEs are substantially higher than the variance they share with any other factor (r^2^ < 0.035 in all cases for Stereotypes, and < 0.431 for Help-Seeking).

However, the criterion was not met for the pair Knowledge and Self-Help. The AVE for Knowledge (0.436) is considerably lower than the shared variance with Self-Help (0.676). Reciprocally, the AVE of Self-Help (0.631) is also lower than its shared variance with Knowledge. This finding does not support discriminant validity between Knowledge and Self-Help according to this criterion, providing stronger evidence of the high empirical overlap between the two constructs.

Finally, as the third and most rigorous approach, the Heterotrait–Monotrait ratio (HTMT) was calculated to evaluate discriminant validity (Henseler et al., 2015). According to this criterion, values must be below the conservative threshold of 0.85 to confirm discriminant validity.

The results of this analysis are presented in Table 6. The highest HTMT value was observed in the relationship between the factors Knowledge and Self-Help (0.820). Since this value, as well as all others in the matrix, is clearly below the strict 0.85 threshold, this analysis provides strong evidence supporting discriminant validity among the four constructs of the model.

In conclusion, although the Fornell–Larcker criterion suggested problematic overlap between Knowledge and Self-Help, the other two approaches—the analysis of confidence intervals and, more conclusively, the HTMT criterion—support the empirical distinction among the four factors.

### 3.1. Item Analysis and Test Information Function (TRI)

#### 3.1.1. Knowledge Subscale

To analyze in detail the item properties of the Knowledge subscale, a Graded Response Model (GRM) was fitted. Table 7 presents the discrimination parameters (α) and the four difficulty thresholds (β) for each of the 12 items that comprise this factor. The discrimination parameter (α), which indicates an item’s ability to differentiate among individuals with varying levels of knowledge, showed considerable variability. Most items demonstrated high to very high discrimination (values of α > 1.5), with items i24 (α = 3.282) and i25 (α = 3.300) standing out as psychometrically excellent indicators.

On the other hand, items i22 (α = 0.713), i02 (α = 0.767), and i03 (α = 0.778) showed more modest, though still acceptable, discrimination. The analysis of the threshold parameters revealed an important finding. For five items (i09, i14, i16, i24, and i25), it was not possible to estimate the upper thresholds. This is due to a ceiling effect in responses: the content of these statements (e.g., “The earlier mental disorders are identified and treated, the better”) generated such strong consensus among participants that the vast majority chose the highest response option (“Strongly agree”).

With very few or no responses in intermediate or lower categories, the model lacks sufficient information to estimate the exact point on the latent trait where transitions to these upper categories would occur. This result reveals an informative feature of these items: they are “easy to endorse” for this population of health and education students.

Figure 3 presents the Item Characteristic Curves (ICCs) for this factor. A visual analysis of the curves confirms these findings. Items with high discrimination, such as i24 and i25, show very steep curves, indicating that they are highly precise in distinguishing among participants. In contrast, items with lower discrimination, such as i22, exhibit flatter curves. Moreover, the ICCs illustrate the ceiling effect: for items such as i09 or i14, the curve for the highest response category (P5) dominates early along the trait continuum, confirming that these are easy items for this population and that they present limited ability to differentiate at higher levels of the construct.

To assess the measurement precision along the continuum of the latent trait, the Test Information Function (TIF) was generated for each factor. Figure 4 presents the results for the Knowledge subscale. The figure displays the TIF (green curve, left axis), which indicates the amount of psychometric information the subscale provides at different levels of knowledge, along with the Standard Error of Measurement (SE) curve (red curve, right axis). The background histogram illustrates the distribution of estimated scores for the participants in the sample. As shown in the graph, the scale provides the greatest amount of information around values of θ = −1.5, corresponding to low or medium-low knowledge levels, while the precision decreases significantly for values greater than θ = 1.5. This indicates that the subscale is especially effective in identifying students with limited or average knowledge, but it offers less diagnostic precision for high-knowledge profiles.

The analysis of Figure 4 reveals that the Knowledge subscale is more precise in measuring participants with low and medium levels of the latent trait. The information curve displays two peaks, reaching its maximum at approximately –2.5 and –1.7, which coincides with the area where a significant portion of the sample is concentrated. As the level of the trait increases above the mean (>0), the information provided by the test progressively decreases, and consequently, the standard error of measurement increases. To quantify this observation, the percentage of total test information contributed within different ranges of the construct was calculated. The results show that 75.6% of the total test information lies in the lower range of the trait (between –4 and 0), while only 14.1% is located in the upper range (between 0 and 4). This finding confirms that the subscale is substantially more precise in measuring and distinguishing individuals with low to medium levels of knowledge than those with high or very high levels. This characteristic is consistent with the ceiling effect previously observed in several items of this factor.

#### 3.1.2. Stereotypes Subscale

The Stereotypes subscale proved to be the most psychometrically robust and homogeneous across the entire questionnaire. As shown in Table 8, all items presented exceptionally high discrimination parameters, with values ranging from 3.812 (i06r) to 4.430 (i23r). According to the classification by Baker and Kim (2017), all indicators of this factor fall into the category of very high discrimination. This finding indicates that the four items are extremely effective at differentiating (with great precision) between individuals who hold greater or lesser erroneous beliefs about mental health.

In terms of threshold parameters, it is observed that they are clustered in the mid-to-lower portion of the latent trait continuum (most between −0.5 and +1.0). This suggests that the subscale is particularly informative and precise for assessing individuals with low to moderate levels of stereotypes. Figure 5 presents the Item Characteristic Curves (ICCs) for the four indicators of the Stereotypes factor. A visual analysis of these curves reinforces the findings from the parameter estimates. All curves exhibit very steep slopes, confirming the strong discriminating power of these items. It is evident that the probabilities of selecting each response category shift dramatically over a very narrow range of the trait continuum. Moreover, the curves for the different items are very similar to one another, reflecting the high internal consistency and homogeneity of this subscale. The thresholds are concentrated around the central zone of the trait continuum (approximately between −1.0 and +1.0), indicating that the subscale is extraordinarily precise for assessing individuals with low to moderate levels of stereotypes, which is where the greatest differentiation between response categories occurs.

Figure 6 presents the Test Information Function (TIF) for the Stereotypes subscale. The analysis of the information curve (green line) reveals that this dimension is extraordinarily precise. The TIF reaches a very high peak (above 20) at the center of the trait continuum (≈0.2), indicating that the subscale measures participants with moderate levels of stereotypes with exceptional precision. The background histogram, which displays the distribution of participants, confirms that the information peak coincides with the area where most of the sample is concentrated. Unlike the Knowledge factor, the information is distributed more evenly on both sides of the mean. The range-based information calculation quantifies this observation: 36.8% of the information is located in the −4 to 0 range, and 63.6% is found in the 0 to 4 range. Taken together, the TIF confirms the excellent psychometric quality of the Stereotypes subscale, demonstrating that it is a highly reliable tool for assessing the full spectrum of this construct, with especially high precision at moderate levels of the trait.

#### 3.1.3. Help-Seeking Subscale

Table 9 presents the estimated parameters for the items associated with the Help-Seeking factor under the Graded Response Model (Samejima, 1969). As shown in Table 9, items i08 (α = 3.431) and i29 (α = 3.302) exhibit the highest discrimination, suggesting they are especially effective at distinguishing individuals with different levels of help-seeking. In contrast, item i11 (α = 1.036) shows the lowest discrimination, indicating less precision in differentiation. The β parameters correspond to the difficulty thresholds for each response category. Across all items, the thresholds are negative, which implies that even individuals with low levels of the trait have a probability of selecting higher categories. However, relevant differences are observed: item i11 displays extremely low thresholds (β_1_ = −4.953), indicating that this item is easy in terms of trait demand. In contrast, items such as i08 and i29 show less extreme thresholds (β_1_ ≈ −2), suggesting they require relatively higher levels of help-seeking to reach upper response categories.

In summary, the results show that most items are appropriate for capturing variability in the trait, although their discrimination and difficulty levels differ. Items i08 and i29 are particularly useful for precise trait estimation, while those with very low thresholds (e.g., i11) may be valuable for identifying individuals with very low levels of help-seeking.

Figure 7 shows the Item Characteristic Curves (ICCs) corresponding to the items of the Help-Seeking factor. All items exhibit an orderly pattern of response categories, indicating that the model functions properly and there are no severe misfits. The transitions between categories are progressive, although the width and position of the curves vary across items. Items with high discrimination, such as i08 and i29, show narrow and well-defined curves, concentrated in specific ranges of the latent trait. This confirms their capacity to accurately differentiate between intermediate and high levels of help-seeking. In contrast, items like i11 display flatter and more overlapping curves, reflecting lower discrimination and, therefore, less precision in distinguishing between trait levels.

Regarding difficulty, items such as i11 and i18 show curves shifted toward negative θ values, indicating they are more accessible to individuals with low levels of help-seeking. Conversely, items like i08 and i29 are centered around values closer to zero, implying they require relatively higher levels of the trait to reach upper response categories.

This pattern suggests that the set of items adequately covers the trait continuum, although their contribution varies: items with sharper curves provide more information at intermediate and high levels, whereas those shifted toward negative values are useful for identifying individuals with low help-seeking levels.

Figure 8 presents the Test Information Function (green line) and the Standard Error of Measurement (red line) for the Help-Seeking factor. The analysis reveals that the greatest amount of information is concentrated within the θ range between −4 and 0, where 81.8% of the total information is accumulated. This indicates that the subscale is more precise in assessing low to moderately low levels of help-seeking. In contrast, the interval between 0 and 4 contributes only 11.2% of the information, suggesting a lower capacity to discriminate high levels of the trait. This pattern is reflected in the sharp increase in standard error beginning at positive θ values, implying that estimates at high levels of the trait are less reliable.

In summary, the information function confirms that the subscale is optimized to assess individuals with low tendencies to seek help, offering precise estimates within that range. However, its utility decreases at higher levels of the trait, which may limit its ability to differentiate between individuals with high willingness to seek help. This finding has implications for the interpretation of extreme scores and for the design of instruments intended to cover the entire continuum of the construct.

#### 3.1.4. Self-Help Subscale

Table 10 presents the estimated parameters for the items comprising the Self-Help factor. Regarding discrimination, item i07 shows the highest value (α = 3.197), indicating a high sensitivity to distinguish between individuals with varying levels of self-help. Item i19 displays moderate discrimination (α = 2.342), while i01 exhibits the lowest discriminative capacity (α = 1.778), although it remains acceptable for measurement purposes. These results suggest that i07 is the most informative item within the subscale. With respect to the difficulty thresholds, all items present negative values in the initial categories, indicating that even individuals with low levels of the trait have a probability of selecting higher response options. Item i01 displays the most extreme thresholds (β_1_ = −4.272), making it the easiest item in terms of trait demand. In contrast, i19 includes a positive threshold (β_3_ = 0.059), meaning that selecting the highest category requires a higher level of self-help. This pattern suggests that the subscale covers a broad range of the continuum, although with greater precision at low and intermediate levels.

In summary, the results show that the Self-Help subscale consists of items with adequate discrimination and diverse threshold values, which supports the measurement of the construct across different levels. Nevertheless, the greater concentration of negative thresholds indicates an optimization toward evaluating individuals with low or moderate willingness to engage in self-help.

Figure 9 shows the Item Characteristic Curves (ICCs) corresponding to the items of the Self-Help factor. In general, the curves display an appropriate ordering and progressive transitions between categories, indicating that the model functions correctly. However, notable differences are observed between items. Item i07, which previously showed the highest discrimination, presents narrow and well-defined curves, concentrated in intermediate ranges of the trait. This confirms its ability to accurately differentiate between medium and high levels of self-help. In contrast, item i01 displays flatter curves that are shifted toward negative θ values, indicating that it is easier and contributes information mainly at lower levels of the trait. Item i19 shows an intermediate pattern, with categories extending into positive values, suggesting that this item contributes to measurement at higher levels of self-help, especially in the most extreme category.

In summary, the curves confirm that the subscale covers a broad range of the continuum, although with greater precision at low and intermediate levels. Items such as i07 are the most informative for discriminating in mid-range levels, while i19 contributes at higher levels, and i01 at lower levels. This pattern supports the complementarity of the items in measuring the construct.

Figure 10 presents the Test Information Function (green line) and the Standard Error of Measurement (red line) for the Self-Help factor. The analysis reveals that the greatest amount of information is concentrated within the θ range between −4 and 0, where 83.8% of the total information is accumulated. This indicates that the subscale is more precise in assessing low and moderately low levels of self-help. In contrast, the interval between 0 and 4 contributes only 9.4% of the information, suggesting a lower capacity to discriminate high levels of the trait. This pattern is reflected in the sharp increase in the standard error starting at positive θ values, implying that estimates at high levels of the trait are less reliable. The information function confirms that the subscale is optimized to assess individuals with low self-help tendencies, offering precise estimates in that range. However, its usefulness decreases at elevated levels of the trait, which could limit the ability to differentiate among individuals with high self-help tendencies. This finding has implications for the interpretation of extreme scores and for the design of instruments intended to cover the entire construct continuum.

Taken together, the psychometric analyses provide consistent evidence regarding the internal structure and measurement quality of the MHLq-YA. The results confirm the multidimensional nature of the construct and show that, following the model respecification, a good fit is achieved (CFI = 0.949, TLI = 0.944, RMSEA = 0.068) along with high reliability across all dimensions (ω > 0.75). In addition, the estimates of convergent and discriminant validity reveal that three out of the four factors meet the established criteria, while the relationship between Knowledge and Self-Help requires specific theoretical interpretation. These findings form the basis for analyzing the conceptual and practical implications of the instrument, as well as for discussing its alignment with previous literature and its relevance for mental health literacy intervention needs.

## 4. Discussion

The results obtained in this study provide strong evidence regarding the internal structure and psychometric properties of the MHLq-YA in a Spanish university population.

First, the analyses confirmed that a four-factor correlated model fits the data substantially better than a unidimensional model, supporting the multidimensional nature of the construct. This finding is consistent with the original proposal by Dias et al. (2018) and with prior adaptations in other cultural contexts (Campos et al., 2022; Wu et al., 2023). However, achieving good model fit required specific modifications, including the elimination and reassignment of items, in line with other international validations that have also adjusted the structure to optimize factorial performance (Günaydin et al., 2023; Jumbe et al., 2023). This pattern suggests that while the construct is theoretically stable, its operationalization requires cultural and contextual adaptations to ensure validity (O’Connor et al., 2014).

Regarding convergent validity, three out of the four dimensions exceeded the recommended threshold for Average Variance Extracted (AVE), while the Knowledge factor showed more modest values. This result is consistent with prior studies that highlight difficulties in capturing variability in declarative knowledge items, particularly in populations with high exposure to psychoeducational content (Kutcher et al., 2016). The strong correlation observed between Knowledge and Self-Help (r = 0.822) raises questions about their conceptual independence, though it may be theoretically interpreted as a functional relationship: greater mental health knowledge acts as a precursor to the adoption of self-care strategies (Jorm, 2012). This interpretation is consistent with Jorm’s foundational conceptualization of mental health literacy (Jorm et al., 1997; Jorm, 2012), which explicitly defines “knowledge of effective self-help strategies” as a knowledge-based subcomponent of the construct rather than a fully independent behavioral dimension. From this theoretical standpoint, self-help competencies are, by definition, a specialized subset of declarative mental health knowledge, which offers a conceptual explanation for why these two dimensions are particularly difficult to empirically disentangle. However, from a strict psychometric perspective, the current instrument struggles to differentiate these constructs empirically. Future refinements of the MHLq should prioritize developing highly specific indicators to better disentangle declarative knowledge from actionable self-care behaviors.

This finding has important practical implications, suggesting that interventions aimed at improving mental health literacy should integrate strong psychoeducational components to enhance self-efficacy in managing emotional difficulties. In addition, highlights a vital self-preservation mechanism with direct clinical relevance. Healthcare and educational personnel are highly vulnerable to occupational hazards such as burnout, compassion fatigue, and vicarious trauma due to continuous exposure to patient suffering. Untreated mental health conditions among providers can lead to reduced quality of care, increased medical errors, and emotional exhaustion (Liu et al., 2025). In this context, MHL is not merely an external tool for patient care, but an internal clinical protective factor. Our findings suggest that an enriched understanding of mental health symptoms serves as a cognitive precursor that empowers professionals to implement timely self-help strategies. Clinically, protecting the mental health of the provider is a prerequisite for maintaining the safety of patient care; thus, psychoeducational curricula must explicitly link external diagnostic knowledge with internal self-care competencies.

The analyses based on Item Response Theory (IRT) provide additional insights into the functioning of the instrument. The test information functions revealed that the Help-Seeking and Self-Help subscales are more precise in assessing low to moderate levels of the trait, while their ability to discriminate at high levels is limited. This finding, along with the ceiling effect observed in several items of the Knowledge factor, suggests that the questionnaire is optimized to identify students with limited literacy, which is particularly useful for designing preventive programs targeting this segment.

Nevertheless, the lower precision at higher trait levels highlights the need to develop additional items capable of assessing advanced competencies, especially in professional contexts where high levels of mental health literacy are expected. Overall, the results reinforce the utility of the MHLq-YA as a diagnostic tool in educational settings, but also point to areas for further optimization. Future research should explore the predictive validity of the questionnaire in relation to psychological well-being and help-seeking behaviors, as well as assess its sensitivity to change in longitudinal interventions. Additionally, it would be relevant to develop adapted versions that enhance information at the higher end of the trait continuum, making the instrument applicable to diverse populations, including both clinical and non-clinical contexts.

Furthermore, the Self-Help and Help-Seeking dimensions carry profound implications for workforce sustainability and organizational clinical safety. Healthcare and education professionals experience disproportionately high rates of occupational burnout, secondary traumatic stress, and compassion fatigue. A practitioner who cannot accurately appraise their own psychological distress or who harbors self-stigma regarding help-seeking is significantly more vulnerable to clinical impairment. This impairment directly compromises patient care through increased medical errors, decreased empathy, and premature attrition. Validating an instrument that accurately captures these dimensions provides faculty and hospital administrators with a vital diagnostic tool to implement targeted psychological first-aid training before student’s transition into demanding clinical residencies.

The findings of this study emphasize the importance of assessing mental health literacy in strategic professional profiles, such as future teachers and healthcare personnel. From a clinical standpoint, the exceptional psychometric robustness and measurement precision of the Stereotypes subscale carry significant implications for healthcare environments. Extensive literature confirms that implicit biases and stigmatizing attitudes among healthcare providers constitute a critical barrier to patient recovery, reducing therapeutic trust and delaying treatment adherence (Anisei & Cojocaru, 2025). Because our final model demonstrates that this subscale functions with optimal precision in the moderate ranges of the trait, the MHLq-YA emerges as an invaluable diagnostic tool. It allows institutions to accurately screen for subtle, non-explicit stigmatizing beliefs in future professionals before they enter clinical rotations or classrooms, enabling the design of targeted anti-stigma interventions that directly safeguard future patient-provider relationships. In educational contexts, high literacy facilitates the early identification of emotional distress, promotes the use of support resources, and helps reduce stigma from early ages. Educators are uniquely positioned to observe prolonged behavioral changes in students, making their MHL the essential bridge that connects vulnerable individuals in school settings to professional psychological and clinical referral pathways (UNESCO Teacher Task Force, 2023). Without adequate MHL, this early detection link is severed, significantly delaying necessary professional care. Therefore, having valid and reliable tools to measure this construct in university training contributes to improving the quality of care and promoting institutional environments sensitive to mental health.

### 4.1. Limitations and Future Research Directions

#### 4.1.1. Limitations

This study presents methodological limitations that must be considered when interpreting the results. First, the sample consists entirely of university students, a population that may be particularly susceptible to social desirability bias; knowing they are being evaluated as future health and education professionals, participants might unconsciously adapt their responses to align with perceived social and academic expectations. Second, the specific age category (young adults) restricts the generalizability of these psychometric findings to older or younger populations. Third, the sample is exclusively composed of nursing and education students—individuals who, by the nature of their vocational choices, may possess inherently more empathetic and prosocial personality structures compared to students in other academic disciplines, potentially skewing baseline MHL scores. Fourth, regarding the sample composition, nursing and education students were included and analyzed together within the same study, rather than separately. This combined approach was deliberately chosen because both groups represent the future ‘frontline’ of human services and community care. Validating the MHLq-YA across this combined cohort allowed us to establish the psychometric stability and utility of the instrument for a broader, yet conceptually cohesive, category of future professionals who share a fundamental need for high mental health literacy. Fifth, the use of convenience sampling restricts the representativeness of the sample, which limits the generalizability of the findings to other university and non-university populations. The gender-skewed distribution introduces a potential composition effect that could influence the factorial estimates and the stability of model parameters.

Sixth, although the final analytical sample (N = 416) is adequate for the CFA models estimated in this study, it falls slightly below the N ≈ 500 benchmark typically recommended for accurate item-parameter recovery in multidimensional graded response models (Jiang et al., 2016). Given the moderate length of the final 26-item scale, this limitation is unlikely to have severely compromised the IRT estimates, but replication of the item parameters in an independent, larger sample is recommended. Furthermore, while retained to preserve ecological validity, the inclusion of the 32 multivariate outliers introduces extreme response patterns that further constrain the generalizability of the specific factor structure to broader populations.

Likewise, the cross-sectional design prevents the assessment of the instrument’s temporal stability and sensitivity to change, which are critical aspects for establishing longitudinal validity. In the item analysis, a ceiling effect was identified in several statements within the Knowledge factor, which reduced the estimability of the upper thresholds and concentrated the information function in the low and middle levels of the trait. This pattern suggests that the instrument does not adequately discriminate at the upper end of the construct, which constitutes a limitation for studies aiming to assess advanced literacy.

Moreover, the high correlation between the Knowledge and Self-Help factors raises questions about their conceptual independence, which should be addressed through more stringent discriminant validity analyses and experimental studies. An additional structural limitation concerns the Stereotypes factor, which is exclusively composed of reverse-worded indicators (i06r, i10r, i13r, i23r). Its exceptionally high factor loadings likely reflect a confounding mixture of substantive coherence and wording-method effects. As recent literature on wording effects cautions (Ponce et al., 2022), disentangling substantive trait variance from methodological artifacts is exceptionally challenging when the construct itself (stigmatizing beliefs) inherently aligns with the negative directionality of the items. Furthermore, while these wording effects may partially capture ephemeral response processes, contemporary methodological guidelines strongly emphasize the ongoing utility of retaining reverse items in psychological assessment (Ponce et al., 2023).

#### 4.1.2. Future Research Directions

Future scale iterations should incorporate positively worded stereotype indicators to properly isolate method variance. In addition, more heterogeneous samples in terms of sociodemographic and contextual variables and apply the questionnaire to clinical and non-clinical populations in order to assess factorial invariance should be included in future research. It is recommended to develop additional items that increase information at higher trait levels, particularly in the Knowledge and Self-Help dimensions. Likewise, it would be appropriate to conduct longitudinal studies that allow for the estimation of temporal stability and sensitivity to change, as well as to explore the predictive validity of the instrument in relation to indicators of psychological well-being, help-seeking behaviors, and academic outcomes.

## Figures and Tables

**Figure 1:**
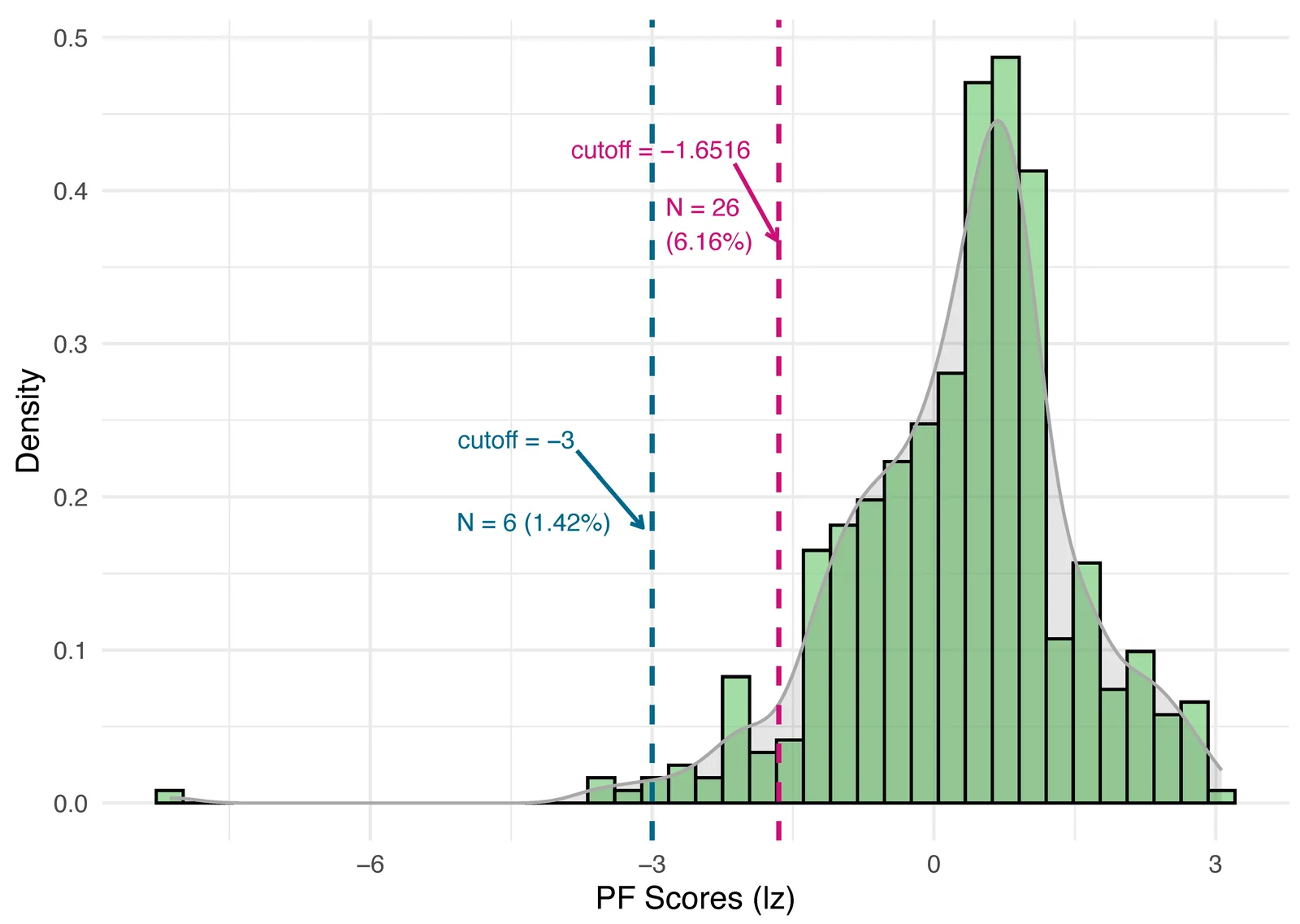
PFScores Distribution.

**Figure 2:**
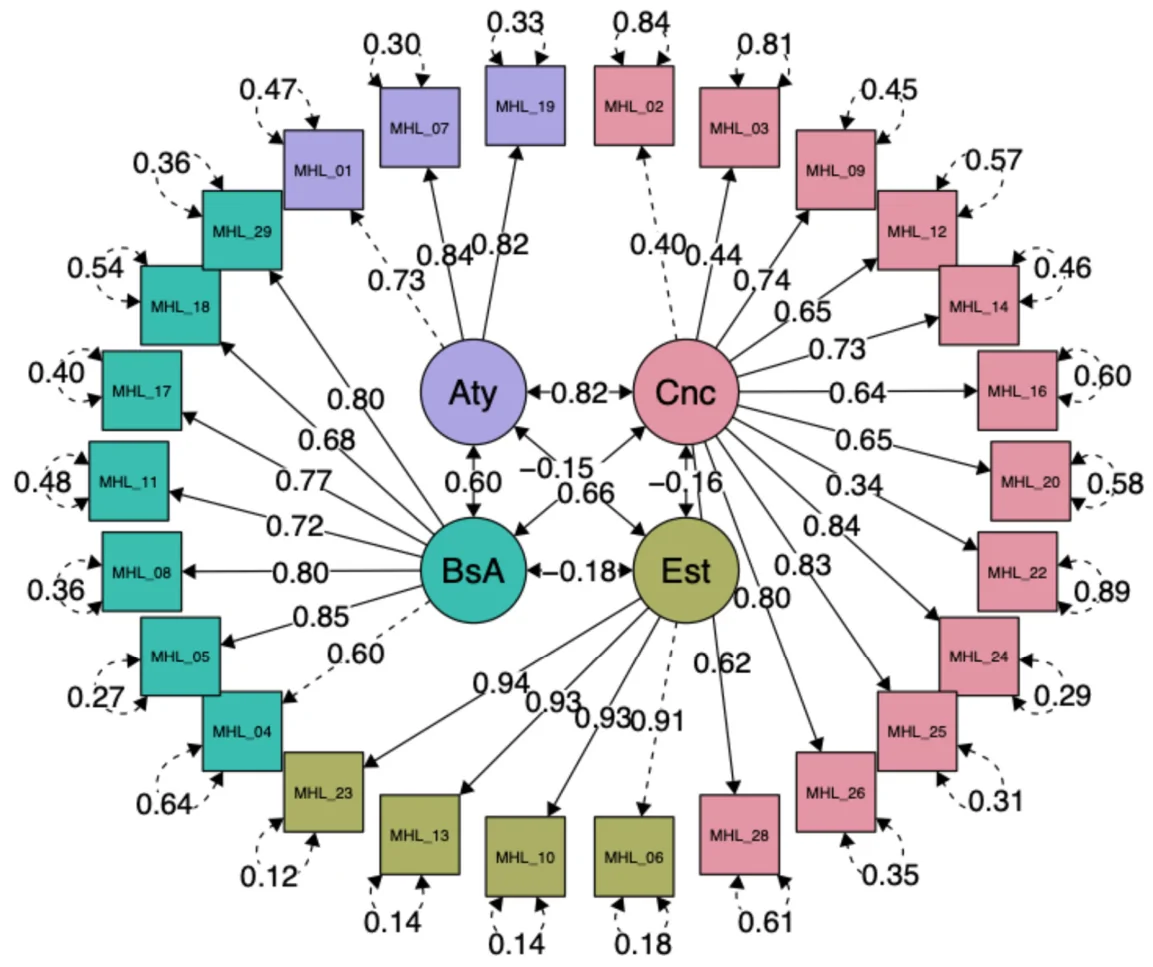
Four-Factor Correlated Model (26 items).

**Figure 3:**
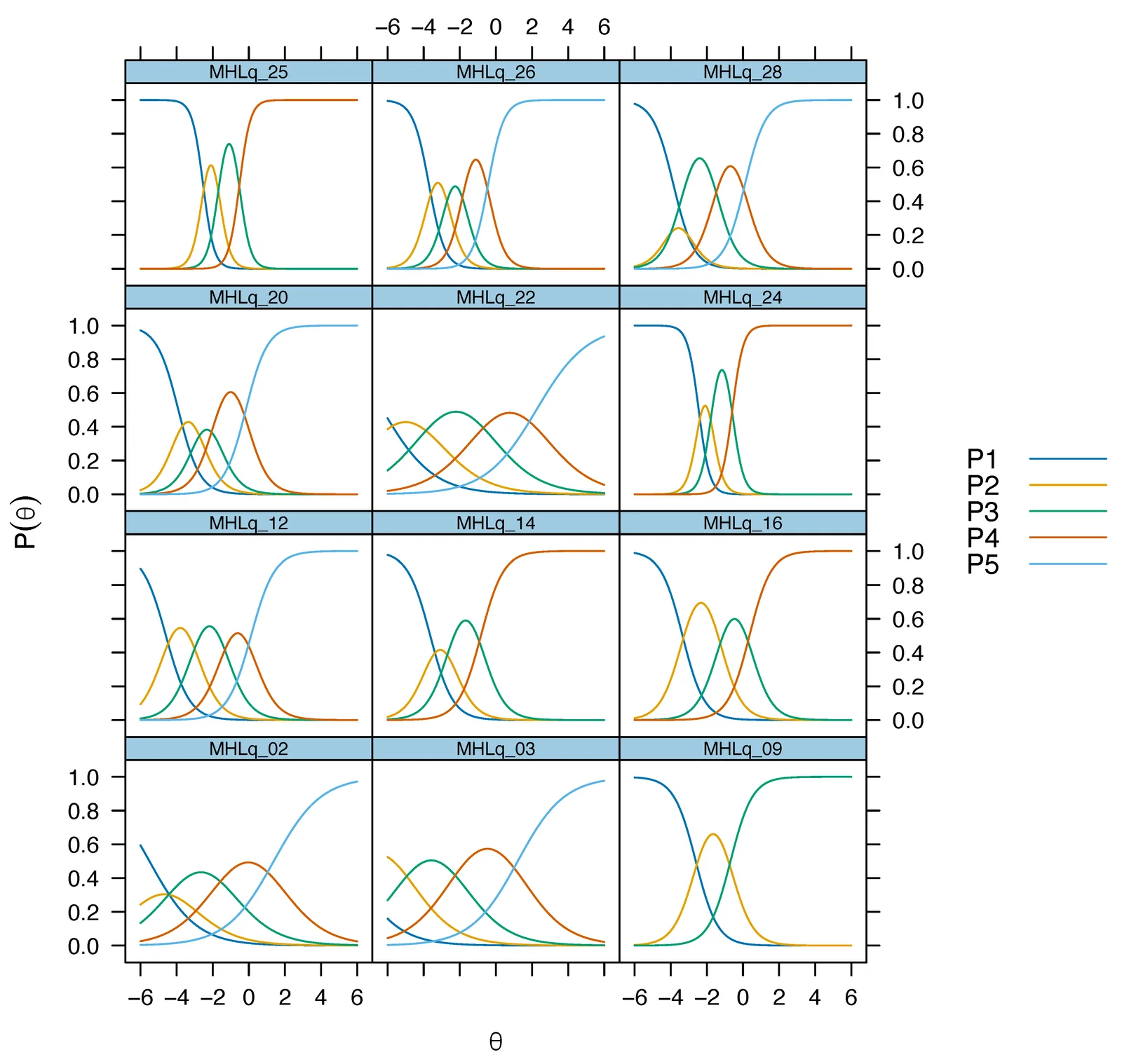
Characteristic Curves from Knowledge Subscale Item.

**Figure 4:**
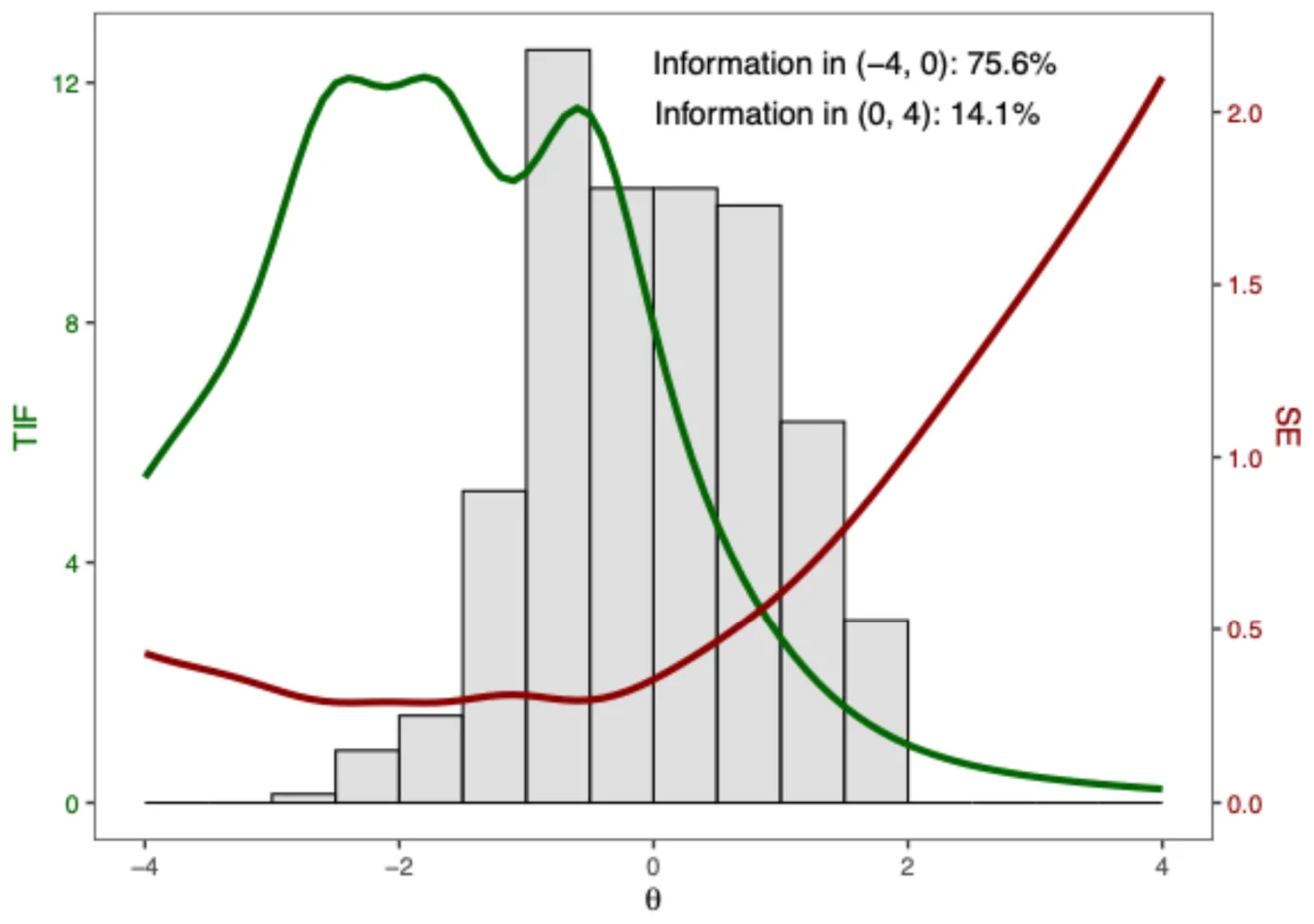
Test Information Function and Standard Error for Knowledge Factor.

**Figure 5:**
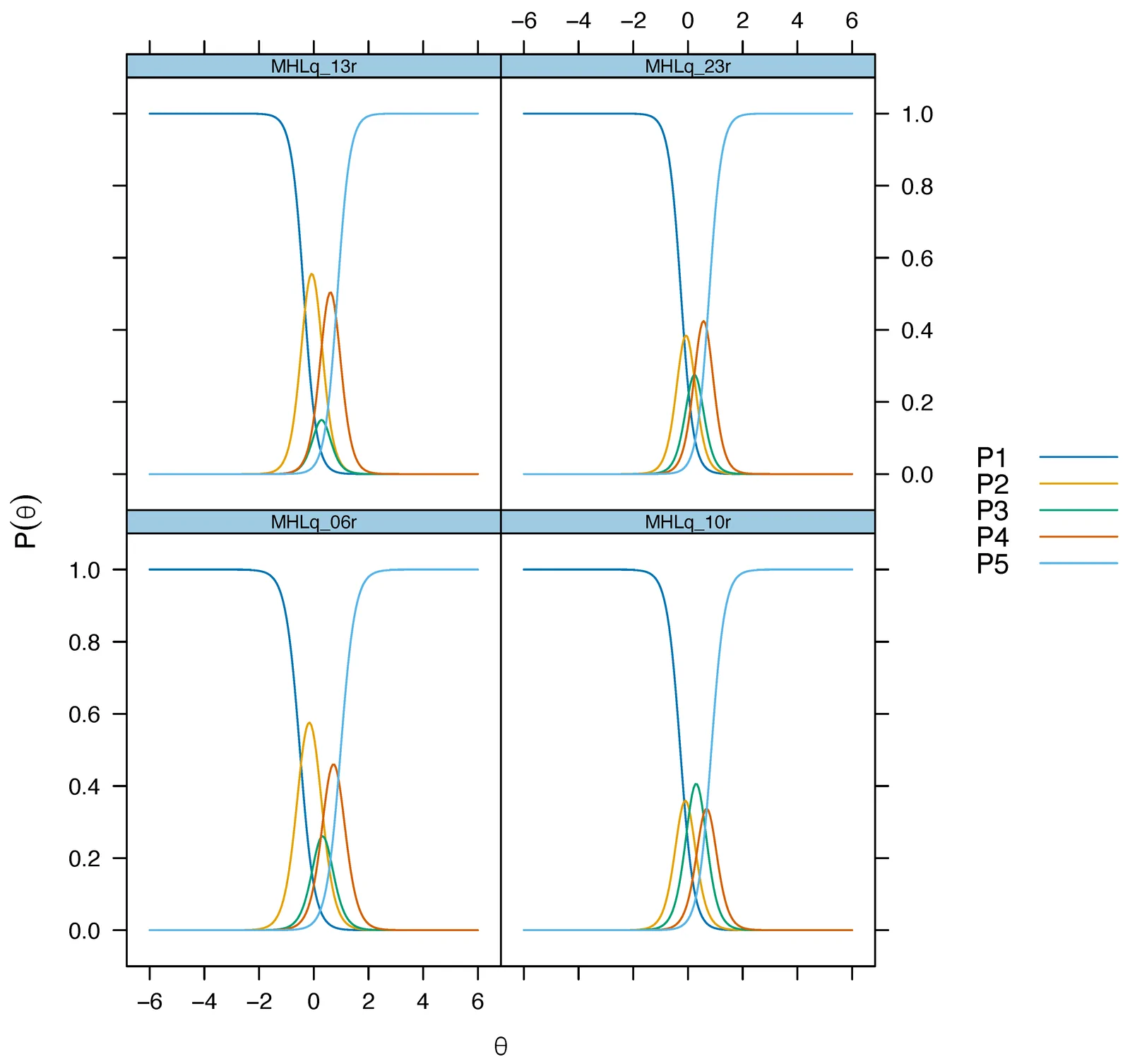
Item Characteristic Curves from Stereotypes Factor.

**Figure 6:**
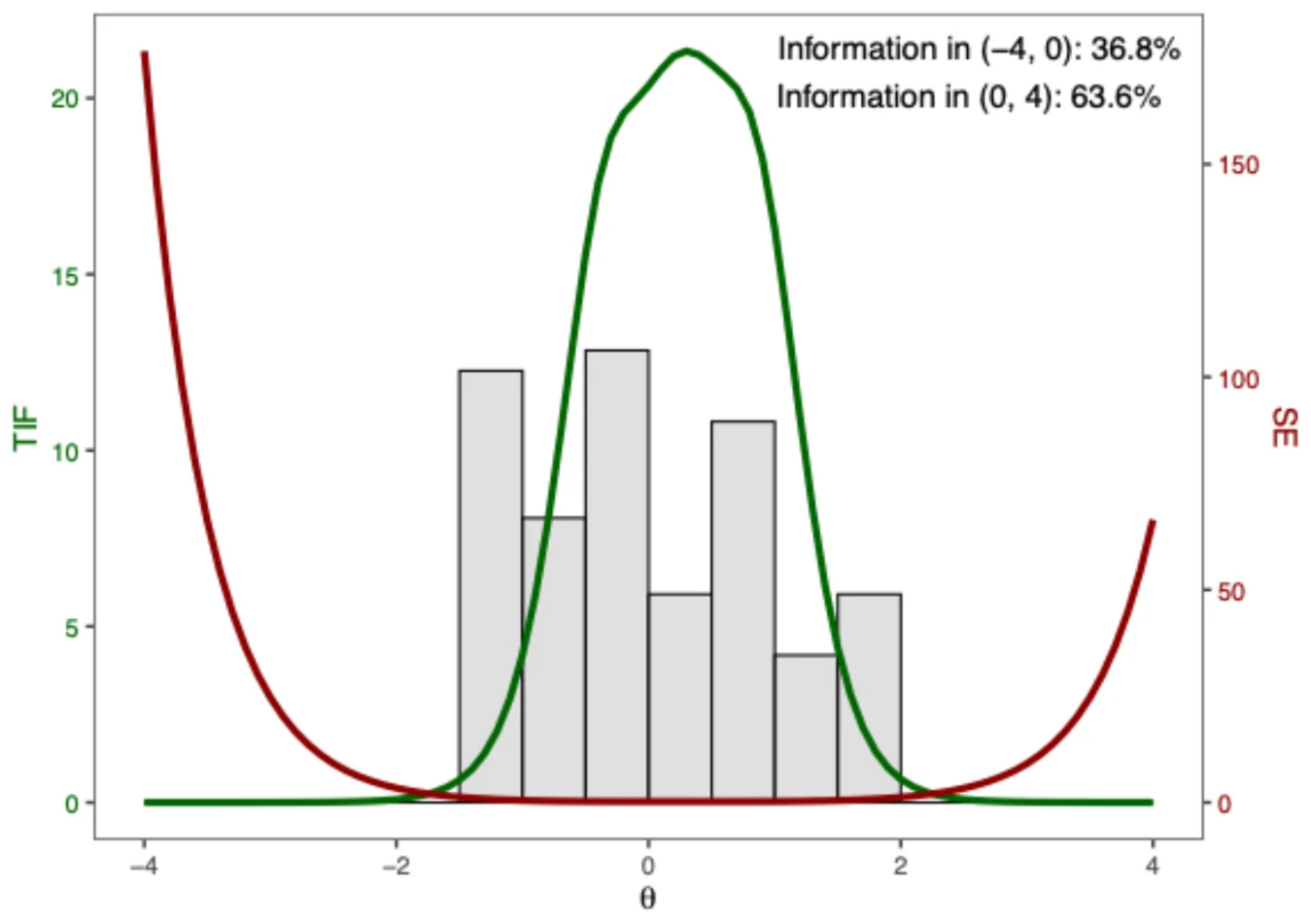
Test Information Function and Standard Error for Stereotypes Factor.

**Figure 7:**
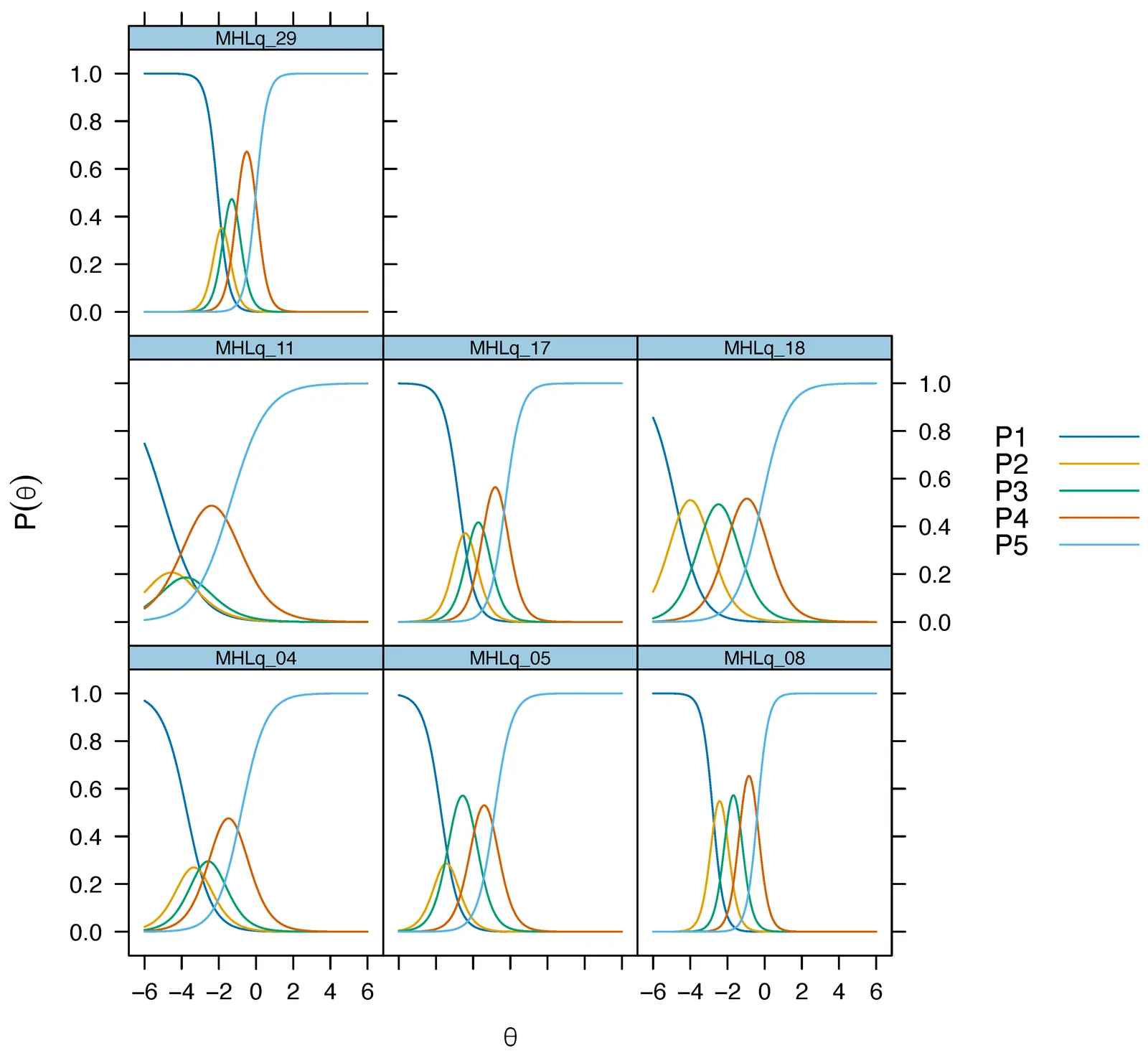
Characteristic Curves Item from Help-Seeking Factor.

**Figure 8:**
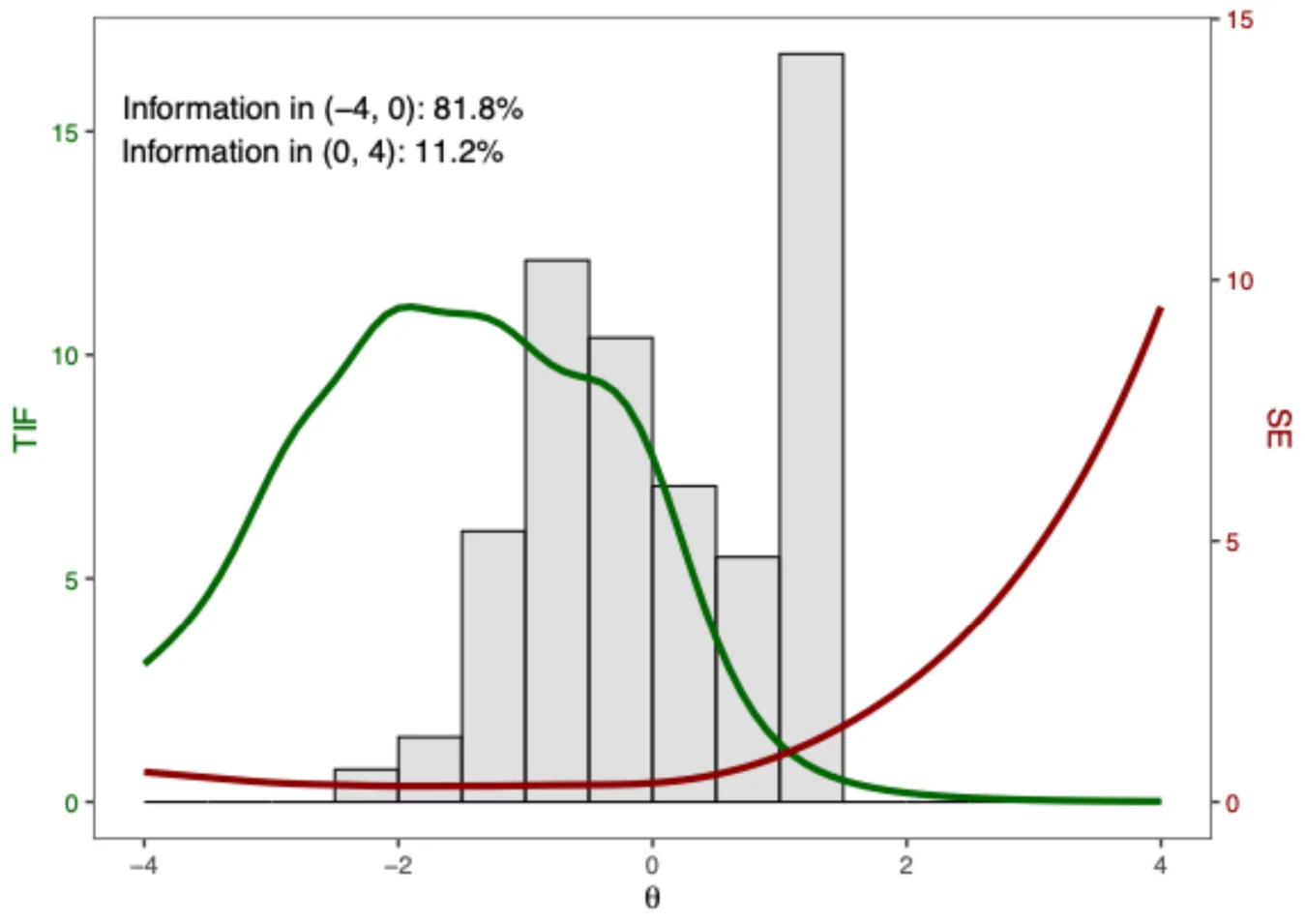
Information Function and Standard Error for Help-Seeking Factor.

**Figure 9:**
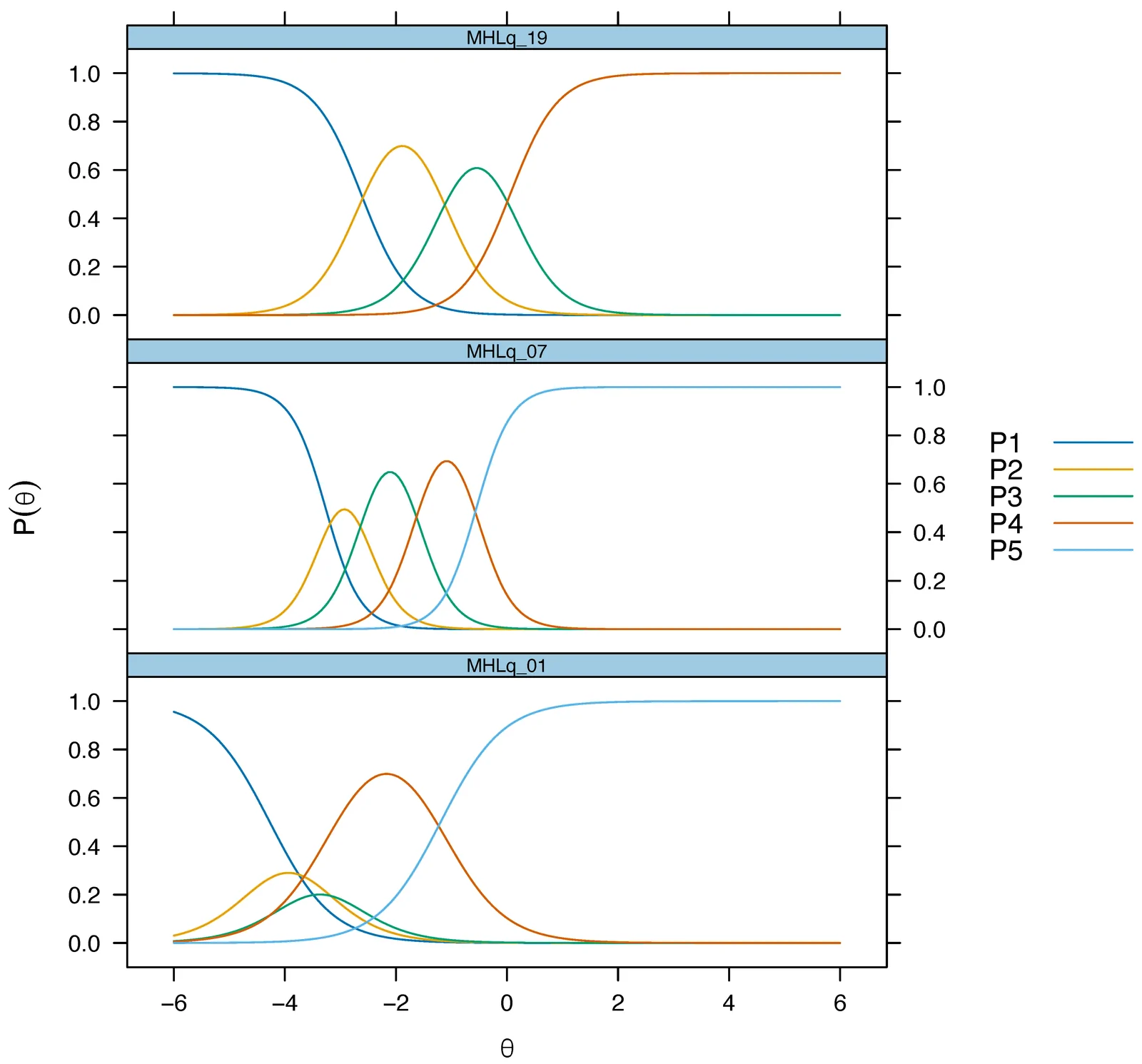
Characteristic Curves form Self-Help Factor Item.

**Figure 10:**
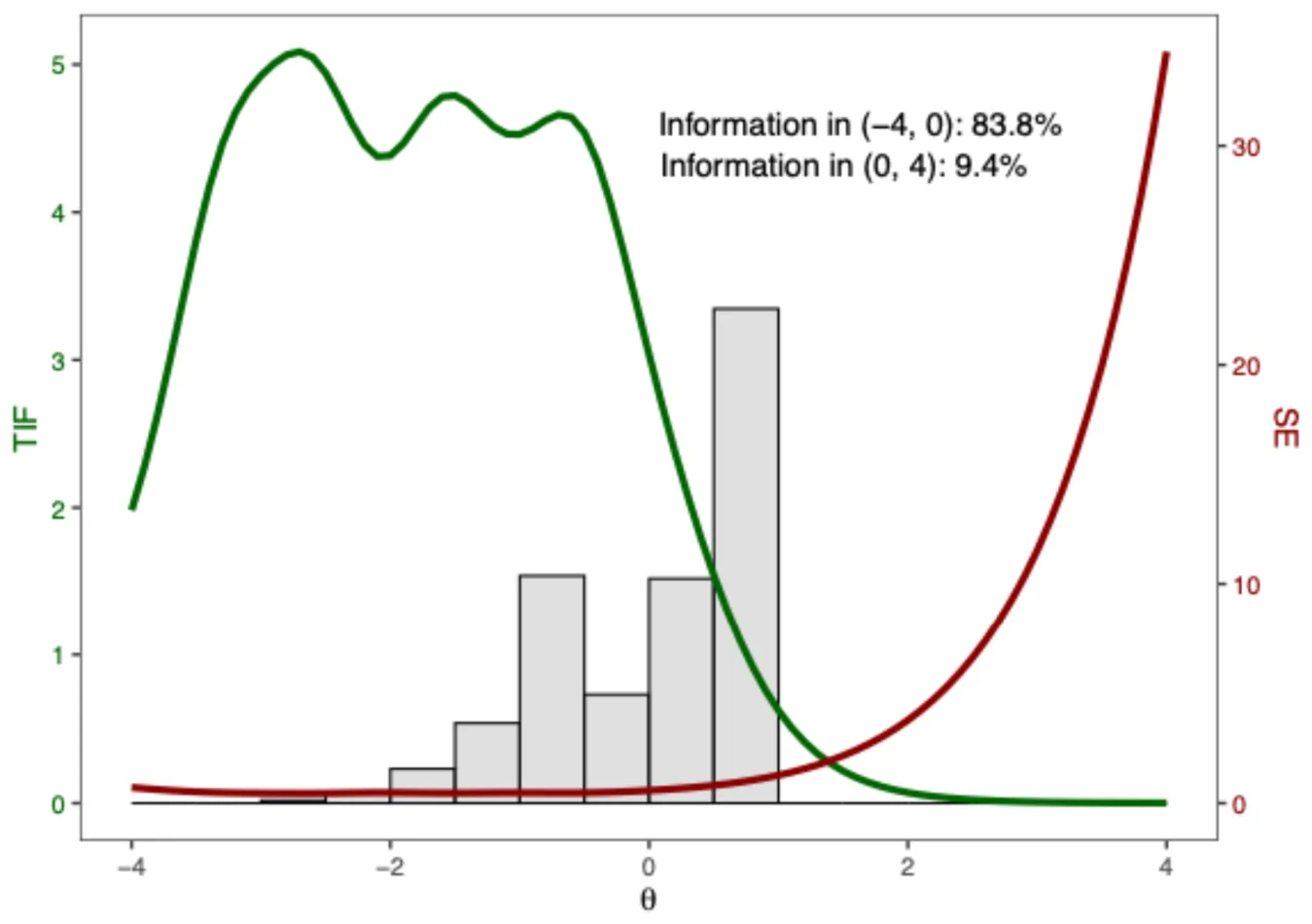
Information Function and Standard Error for Self-Help factor.

**Table 1:** Sociodemographic and Contextual Characteristics of the Initial Sample (N = 425).

Characteristic	Category/Statistic	Value
Age (years)	Mean (DE)	20.32 (2.45)
Gender		
	Females	325 (76.5%)
	Males	100 (23.5%)
Field of Study		
	Early Childhood Education	50 (11.8%)
	Primary Education	169 (39.8%)
	Nursing	206 (48.5%)
Academic Year		
	1º	62 (14.6%)
	2º	191 (44.9%)
	3º	131 (30.8%)
	4º	40 (9.4%)
	Not specified	1 (0.2%)
Province (Grouped)		
	Valladolid	209 (49.2%)
	Salamanca	124 (29.2%)
	Burgos	22 (5.2%)
	Other provinces (n = 24)	70 (16.5%)
	Not specified	0 (0.0%)
Place of Residence		
	City > 500,000 inha.	33 (7.8%)
	City > 100,000 and <500,000 inha.	265 (62.4%)
	City > 20,000 and <100,000 inha.	49 (11.5%)
	Town < 20,000 inha.	77 (18.1%)
	Not specified	1 (0.2%)
Prior Contact with individuals with ID		
	No	196 (46.1%)
	Yes	229 (53.9%)
Religion (Grouped)		
	Catholic	279 (65.6%)
	Other	5 (1.2%)
	No religion	140 (32.9%)
	Not specified	1 (0.2%)
Political Ideology		
	Centrist	249 (58.6%)
	Conservative	90 (21.2%)
	Progressive	85 (20.0%)
	Not specified	1 (0.2%)

Note. Values for categorical variables are n (%). For Age, value is Mean (SD = Standard Deviation).

**Table 2:** Fit Indices and Comparison of Confirmatory Factor Models (N = 416).

Model	χ^2^	gl	CFI	ΔCFI	TLI	ΔTLI	RMSEA [90% CI]	ΔRMSEA	SRMR
Model 1 (Unifactorial)	4719.482	377	0.834	−0.050	0.821	−0.052	0.167 [0.162, 0.171]	0.026	0.242
Model 2 (4 Factors)	3402.627	371	0.884	0.000	0.873	0.000	0.140 [0.136, 0.145]	0.000	0.192
Model 3 (Hierarchical)	3378.479	373	0.885	0.001	0.875	0.002	0.139 [0.135, 0.144]	−0.001	0.197

Note: Δ values represent the difference compared to Model 2 (Four Correlated Factors). CFI = Comparative Fit Index; TLI = Tucker–Lewis Index; RMSEA = Root Mean Square Error of Approximation; SRMR = Standardized Root Mean Square Residual.

**Table 3:** Standardized Parameters of the Final Four-Factor Model (26 items).

Factor	Item	B	SE	z	β	R^2^	*p*
Knowledge	i02	1.000	0.000		0.396	0.157	
	i03	1.098	0.120	9.116	0.435	0.189	<0.001
	i09	1.868	0.194	9.650	0.740	0.548	<0.001
	i12	1.648	0.190	8.658	0.653	0.426	<0.001
	i14	1.847	0.207	8.919	0.732	0.536	<0.001
	i16	1.605	0.184	8.715	0.636	0.405	<0.001
	i20	1.640	0.174	9.431	0.650	0.423	<0.001
	i22	0.855	0.138	6.187	0.339	0.115	<0.001
	i24	2.124	0.221	9.597	0.842	0.709	<0.001
	i25	2.098	0.220	9.528	0.831	0.691	<0.001
	i26	2.028	0.218	9.298	0.804	0.646	<0.001
	i28	1.570	0.178	8.823	0.622	0.387	<0.001
Stereotypes	i06r	1.000	0.000		0.906	0.822	
	i10r	1.022	0.018	57.812	0.926	0.858	<0.001
	i13r	1.022	0.021	49.671	0.927	0.859	<0.001
	i23r	1.036	0.019	54.625	0.939	0.882	<0.001
Help-Seeking	i04	1.000	0.000		0.597	0.356	
	i05	1.427	0.119	12.023	0.852	0.726	<0.001
	i08	1.336	0.101	13.282	0.797	0.636	<0.001
	i11	1.213	0.111	10.958	0.724	0.524	<0.001
	i17	1.298	0.109	11.869	0.775	0.600	<0.001
	i18	1.138	0.088	12.870	0.679	0.461	<0.001
	i29	1.338	0.107	12.514	0.799	0.638	<0.001
Self-Help	i01	1.000	0.000		0.727	0.529	
	i07	1.148	0.089	12.935	0.835	0.698	<0.001
	i19	1.123	0.086	13.083	0.817	0.667	<0.001

Note. B = Unstandardized factor loading; SE = Standard error; β = Standardized factor loading; R^2^ = Explained item variance.

**Table 4:** Correlations Between Latent Factors and Their 95% Confidence Intervals.

Factor 1	Factor 2	Correlation (r)	95% CI Lower	95% CI Upper
Knowledge	Stereotypes	−0.164	−0.258	−0.069
Knowledge	Help-Seeking	0.655	0.590	0.720
Knowledge	Self-Help	0.822	0.769	0.875
Stereotypes	Help-Seeking	−0.183	−0.283	−0.084
Stereotypes	Self-Help	−0.151	−0.274	−0.028
Help-Seeking	Self-Help	0.600	0.516	0.684

**Table 5:** Fornell–Larcker Criterion Results for Discriminant Validity.

	Knowledge	Stereotypes	Help-Seeking	Self-Help
Knowledge	**0.436**	0.027	0.430	0.676
Stereotypes	0.027	**0.855**	0.034	0.023
Help-Seeking	0.430	0.034	**0.563**	0.359
Self-Help	0.676	0.023	0.359	**0.631**

Note. Diagonal values (in bold) represent the Average Variance Extracted (AVE) of each factor. Off-diagonal values represent shared variance (squared correlation) between pairs of factors.

**Table 6:** Heterotrait–Monotrait Ratio (HTMT) Matrix.

	Knowledge	Stereotypes	Help-Seeking	Self-Help
Knowledge	—	0.167	0.658	0.820
Stereotypes	0.167	—	0.191	0.154
Help-Seeking	0.658	0.191	—	0.627
Self-Help	0.820	0.154	0.627	—

Note. Values below the conservative threshold of 0.85 are considered evidence of discriminant validity.

**Table 7:** Item Parameters for the Knowledge Factor (GRM Model).

Item	α	β_1_	β_2_	β_3_	β_4_
i02	0.767	−5.503	−3.866	−1.438	1.378
i03	0.778	−8.133	−5.004	−2.148	1.208
i09	1.653	—	—	−2.613	−0.691
i12	1.534	−4.593	−2.996	−1.362	0.122
i14	1.597	—	−3.635	−2.527	−0.829
i16	1.672	—	−3.347	−1.297	0.358
i20	1.675	−3.903	−2.809	−1.846	−0.170
i22	0.713	−6.278	−3.713	−0.711	2.243
i24	3.282	—	−2.451	−1.740	−0.589
i25	3.300	—	−2.533	−1.667	−0.517
i26	2.277	−3.708	−2.723	−1.784	−0.433
i28	1.751	−3.853	−3.292	−1.502	0.110

Note. α = Item discrimination parameter; β = Threshold/difficulty parameters. Missing values (—) denote non-estimable thresholds due to zero empirical frequencies in the lower response categories (ceiling effect). The reported β parameters have been aligned to reflect their true category transitions.

**Table 8:** Item Parameters for the Stereotypes Factor (GRM Model).

Item	α	β1	β2	β3	β4
i06r	3.812	−0.508	0.181	0.461	0.984
i10r	4.081	−0.282	0.087	0.509	0.853
i13r	4.352	−0.362	0.215	0.354	0.864
i23r	4.430	−0.256	0.110	0.365	0.775

Note. α = Item discrimination parameter; β = Threshold/difficulty parameters.

**Table 9:** Item Parameters for the Help-Seeking Factor (GRM Model).

Item	α	β1	β2	β3	β4
MHLq_04	1.505	−3.713	−2.978	−2.169	−0.794
MHLq_05	2.140	−3.723	−3.172	−1.958	−0.854
MHLq_08	3.431	−2.781	−2.062	−1.302	−0.389
MHLq_11	1.036	−4.953	−4.146	−3.422	−1.369
MHLq_17	2.372	−2.755	−2.097	−1.348	−0.270
MHLq_18	1.452	−4.775	−3.224	−1.738	−0.164
MHLq_29	3.302	−2.065	−1.618	−0.995	−0.007

Note. α = Item discrimination parameter; β = Threshold/difficulty parameters.

**Table 10:** Item Parameters for the Self-Help Factor (GRM Model).

Item	α	β1	β2	β3	β4
MHLq_01	1.778	−4.272	−3.600	−3.143	−1.195
MHLq_07	3.197	−3.267	−2.589	−1.621	−0.551
MHLq_19	2.342	—	−2.626	−1.147	0.059

**Note.** α = Item discrimination parameter; β = Threshold/difficulty parameters. Missing values (—) denote non-estimable thresholds due to zero empirical frequencies in the lower response categories (ceiling effect). The reported β parameters have been aligned to reflect their true category transitions.

## Data Availability

The data that support the findings of this study are available from the corresponding author upon reasonable request.
